# The impact of free trade port construction on regional import and export: Evidence from Hainan

**DOI:** 10.1371/journal.pone.0328875

**Published:** 2025-08-13

**Authors:** Zhenyu Zhao, Zhongliang Guan, Zhanliang Liu, Xiang Xie

**Affiliations:** School of Economics and Management, Beijing Jiaotong University, Beijing, China; Szkoła Główna Handlowa Warsaw School of Economics, POLAND

## Abstract

The construction of the Hainan Free Trade Port (HFTP) represents one of the most advanced regional development models explored by China. This study clarifies the concept of the free trade port and analyzes it from a trade perspective. Our empirical results show that the construction of the free trade port has significantly increased trade volumes, enhanced the diversification of products, and reduced the volatility of exports. A marked short-term surge in export volumes was observed immediately after the policy’s implementation. Moreover, the effects of the free trade port’s construction on trade are heterogeneous when viewed through the lens of product categorization.

## 1. Introduction

Amid global economic shocks from the COVID-19 pandemic, the Russia-Ukraine conflict, inflation, monetary tightening, and widespread debt issues [1,2], global trade is facing significantly increased uncertainty. Tightening financial conditions have restricted trade financing, contributing to a slowdown in global trade growth. As developing countries grapple with export challenges and demand in developed countries weakens [3], identifying new drivers of global trade and enhancing international trade cooperation have become especially crucial. As a major player in product trade, China needs to address the challenges posed by reduced competitiveness in international markets. Deepening reforms and expanding openness are essential for the vitality of the global economy.

Smooth trade relies on a robust institutional foundation. Reasonable institutional design can reduce transaction costs [4] and minimize informal trade barriers [5], thereby enhancing international exchanges. Therefore, comprehensive institutions are key to fostering trade growth and reducing income disparities between countries and regions [6]. Studies have shown that trade regimes in special economic zones such as free zones, free ports, and free trade areas can positively impact regional growth, attract foreign direct investment (FDI), and promote trade liberalization [7–10]. More specifically, Special Economic Zones not only enhance firm-level export performance, promote industrial agglomeration, and drive institutional reforms [8], but also facilitate e-commerce flows, all of which contribute to broader trade globalization [11].

On June 1, 2020, China officially launched its first free trade port under the socialist system (Hainan Free Trade Port, HFTP) in Hainan Province, marking the first large-scale economic zone deep reform implemented province-wide in China. Through reforming or amending relevant policies, administrative regulations, and departmental rules, the central government aims to quickly grant high autonomy to the HFTP, transforming it into a highly liberalized and influential international trade port, thereby further expanding China’s reform and opening-up achievements.

Although Hainan Province enjoys a unique coastal location, serving as China’s key maritime gateway to Southeast Asia, South Asia, the Middle East, Africa, and Europe, its levels of import and export trade and economic development remained significantly lower than other coastal regions prior to the establishment of the HFTP. The launch of the HFTP marked unprecedented policy support from the central government of China, personally planned and deployed by President Xi Jinping, emphasizing the significance of the free trade port’s construction. In Special Economic Zones (SEZs), particularly, the level of imports and exports is one of the key indicators of development success. This study aims to explore the specific impacts of the HFTP on Hainan’s trade, fill research gaps, and discuss the feasibility of establishing free trade port policies in developing countries, providing empirical evidence with quarterly product trade data.

In existing SEZ research, most scholars analyze the trade or economic performance of multiple cities or provinces impacted by policies from a macro perspective or focus on the response of industries or businesses to SEZ policies [8,12,13]. However, achieving significant regional economic or trade growth is challenging for countries that are not underdeveloped, despite these policies generally having indirect positive effects. This phenomenon is primarily due to two reasons: first, international trade is influenced by various complex factors, making it difficult to achieve significant growth in a large region in a short time; second, nations typically focus on coordinated regional development and do not provide excessive policy support and resource allocation to a single large area. Hence, most SEZ policies are limited to specific parts of a province or city’s ports, and large-scale SEZ policies are often too broad to have significant effects. This study leverages the large-scale policy impact of HFTP on the entire island of Hainan, focusing on specific products for imports and exports, offering a more micro-level perspective, revealing the dynamics and trends of segmented product trade, thereby providing a more detailed assessment of how policies specifically affect the import and export of various products. The study utilizes quarterly panel data of 2111 six-digit HS trade products from six coastal provinces of China from the first quarter of 2015 to the first quarter of 2024, employing Difference in Differences (DID) and Regression Discontinuity Design – Difference in Differences (RD-DD) models to empirically evaluate the impact of HFTP construction on product trade.

The main innovations of this study are as follows: First, this research contributes to the existing literature on free trade zones by focusing on one of the core objectives of the Hainan Free Trade Port (HFTP)—trade. While previous studies on the HFTP remain limited and have primarily examined non-economic dimensions such as environmental regulation and medical legislation [14,15]. Second, by analyzing trade volume, product diversification, and volatility, this paper comprehensively assesses the impact of the HFTP on product trade in Hainan Province and compares the effects on imports versus exports. Additionally, this study employs different models to assess the long-term and short-term effects of the HFTP. Third, the research drills down to the level of trade products, discussing the heterogeneous impacts on import and export outcomes across more detailed industry sectors.

The rest of this paper is organized as follows: Section 2 introduces the policy background and posits research hypotheses; Section 3 details the research design and data; Section 4 discusses the DID regression and RD-DD results; Section 5 conducts robustness tests; Section 6 analyzes heterogeneity; the final section summarizes the conclusions and offers policy recommendations.

## 2. Background and theoretical hypothesis

### 2.1. Hainan free trade port background

Special Economic Zones (SEZs) have played a significant role globally. By 2018, there were nearly 5,400 free trade zones worldwide, with 42.6% specifically categorized as free trade zones [16]. SEZs, which include free trade zones, export processing zones, industrial parks, and free zones, are collectively recognized as modern developments in traditional regional economic structures [16–18]. These zones offer a range of incentives, including fiscal, regulatory, and infrastructure support [16], which have spurred industrial production, economic growth, and attracted investments [19,20]. However, the Hainan Free Trade Port (HFTP) differs in important ways from conventional SEZs. Hainan was first designated as a special economic zone in 1988 and became a pilot free trade zone in April 2018. On April 13, 2018, the Chinese government formally announced the plan to explore the establishment of a free trade port on the basis of the free trade zone. The full-scale construction of the HFTP officially began on June 1, 2020, with the release of the “Master Plan for the Construction of the Hainan Free Trade Port.” This plan set two major policy objectives: the first is to promote trade and investment liberalization and facilitation, improve the efficiency of factor flows, and achieve early policy results under effective supervision; the second is to build a world-class free trade port by 2035, featuring a mature legal system, a modern industrial structure, and a globally competitive open economy.

There is considerable ambiguity in the global definition of “free zones,” as various countries adopt different institutional models and policy scopes under this label [7]. China considers highly developed cases such as Singapore, Hong Kong, and Dubai as benchmarks for its free trade port development, despite fundamental differences in their historical trajectories and institutional arrangements. The Hainan Free Trade Port (HFTP), launched in June 2020, represents a distinct evolution from earlier free trade zones and special economic zones (SEZs) implemented across China. While sharing core features such as duty exemptions and trade facilitation, the HFTP is characterized by deeper and broader reforms, as well as a more comprehensive policy mandate. It is best understood as an unconventional, large-scale SEZ reform initiative, strategically advanced by the central government, potentially making it the second most economically liberal zone in China after Hong Kong.

Compared with other free trade zones in China, HFTP exhibits deeper, broader, and more centralized policy implementation. First, it applies uniformly across the entire province of Hainan, while other free trade zones typically cover only designated sub-areas within a city or region [21,22]. Second, the HFTP is directly guided and administered by the central government, with strategic oversight at the highest political level, whereas other FTZs are often more locally governed with provincial authorities playing a larger role in policy design. Third, while both FTZs and HFTP aim to attract investment and promote trade facilitation, the HFTP is envisioned as a comprehensive upgrade that positions Hainan as a major global trade and logistics hub. It expands beyond traditional free trade zone policies by offering zero-tariff treatment on a wider range of products, introducing stronger financial liberalization measures, and supporting large-scale infrastructure and institutional development initiatives.

### 2.2. Theoretical hypothesis

By analyzing the policy documents of HFTP, we have identified that the policy provides multifaceted support and advantages for the region’s imports and exports, mainly reflected in the following four aspects.

The first factor is tax incentives. The HFTP has implemented a series of tax incentives, including reducing corporate income tax rates, offering personal income tax benefits, exempting certain imported products from tariffs, and simplifying the tax refund process. These policies directly reduce the operational costs for businesses and the import costs for products, thereby enhancing the market attractiveness of Hainan [23,24]. Additionally, tax policies are related to the larger fiscal spaces of developing countries, with these positive impacts being particularly pronounced in the least developed areas [25]. Given Hainan’s relatively low level of development among all coastal regions, the construction of the HFTP makes the effects of tax incentives more pronounced.

The second key factor is the simplification of trade procedures and enhancement of logistics efficiency. The HFTP has effectively reduced trade barriers by simplifying customs procedures and improving clearance efficiency. Additionally, transportation efficiency has been enhanced through duty-free fuel policies and the strengthening of transportation infrastructure. More efficient trade procedures and logistics can shorten the turnover cycle for business products, reduce the time and cost of cross-border trade, and increase the transparency of the trading process, thereby boosting trust and promoting the development of imports and exports [26].

The third factor is market liberalization and improvements in the investment environment. The HFTP has fewer restrictions on foreign capital, providing a more relaxed environment for foreign investors and businesses to invest and operate. HFTP employs a negative list approach to restrict specific industries, while the central government has granted Hainan more authority to reduce the negative list for foreign investments, thus expanding the scope of foreign investment. Furthermore, by improving laws and regulations, strengthening infrastructure, and enhancing public services [27], HFTP has optimized the investment environment, attracting significant foreign investment, which has stimulated industry and upgraded infrastructure, thereby promoting the development of import and export trade [28].

The fourth factor is support from industrial policies. HFTP provides policy support for key areas such as high-tech industries, tourism, and modern service industries, offering more conveniences and incentives to relevant businesses and encouraging industrial upgrading and economic diversification. These industry support policies have not only attracted the development of businesses in relevant sectors but have also promoted the import and export activities of these industries [29].

The volume of imports and exports is an important indicator of a region’s degree of economic openness and the prosperity of international trade activities. This indicator not only reflects the region’s participation in the global economy but also serves as a crucial factor in assessing the health and growth potential of the region’s economy. For the HFTP, an increase in the volume of imports and exports directly demonstrates its enhanced competitiveness and attractiveness in international trade, which helps increase local tax revenue, create job opportunities, and promote the upgrading and transformation of local industries. Based on these analyses, we propose the following Hypothesis 1 and Hypothesis 2:

**H1**. The construction of the Hainan Free Trade Port has positively influenced the volume of export trade in Hainan Province.

**H2**. The construction of the Hainan Free Trade Port has positively influenced the volume of import trade in Hainan Province.

Considering the importance of diversification in import and export trade for the health and sustainability of trade, we focus on the necessity of trade diversification. Trade diversification, involving the types and quantities of imported and exported products, helps reduce dependence on a single market or product while enhancing an economy’s resilience to external shocks.

The development of new industries is not isolated but builds on the region’s existing capabilities and resources. This path-dependent strategy of industrial diversification has been globally validated [30]. Some studies have found that countries often develop comparative advantages in new product areas related to their existing export products [31]. Sometimes, regions may also venture into new fields unrelated to existing industries for diversification [32]. The key to unrelated diversification lies in cross-regional cooperation, which can bring in knowledge, technology, and financial resources that are lacking locally [33,34]. Such cooperation might include trade interactions, labor mobility, investment flows, and joint research and development projects [33–35]. HFTP enhances these cross-regional collaborations through institutional innovation. Based on these observations, we further propose Hypothesis 3 and Hypothesis 4 regarding trade diversification.

**H3**. After the establishment of the free trade port, Hainan Province has increased the diversification of its export trade.

**H4**. After the establishment of the free trade port, Hainan Province has increased the diversification of its import trade.

The impact of trade liberalization on macroeconomic volatility is an important topic in macroeconomic development [36–38]. The construction of the HFTP might also influence the volatility of product imports and exports. On one hand, the establishment of the HFTP signifies more stable development policies for Hainan, where policy stability facilitates trade stability [1], and improved logistics policies contribute to a more stable supply chain, thus enhancing trade stability. On the other hand, rapid development in import and export activities, particularly the emergence of new industries and the phasing out of old ones, could increase the volatility of imports and exports. Based on these insights, we propose Hypothesis 5 and Hypothesis 6.

**H5**. After the establishment of the free trade port, Hainan Province has reduced the volatility of its export trade.

**H6**. After the establishment of the free trade port, Hainan Province has reduced the volatility of its import trade.

## 3. Research design

### 3.1. Data

The “Harmonized” HS code system integrates the “Customs Cooperation Council Nomenclature” (CCCN) and the United Nations “Standard International Trade Classification” (SITC), forming a multifunctional international trade commodity classification system. This study spans from the first quarter of 2015 to the first quarter of 2024, focusing on quarterly import and export data using 6-digit HS codes. It selects the top 1107 products by export volume and the top 1004 products by import volume from Hainan Province as research samples. These products account for more than 99.8% of Hainan’s total import value and more than 99.9% of its export value. Descriptive statistics for the main variables of imports and exports are presented in Tables 1 and 2.

**Table 1:** Descriptive statistics of variables: Export.

Variable type	Name	N	Mean	Std	Min	Max	Level
Outcome variables	lnEx	245,754	12.278	6.294	0	23.598	Province × Product × Time
	ExDensity	17,136	11.791	20.170	1	137	Province × Industry (2-digit) × Time
	VarEx	104,008	1.756	6.271	0.001	85.379	Province × Product × Time
Explanatory variable	DTreat	245,754	0.0675	0.251	0	1	Province × Time
Control variables	LnGdp	245,754	9.686	1.114	6.734	11.818	Province × Time
	LnWIm	245,754	24.954	1.531	18.011	27.668	Industry (2-digit)× Time
	LnCEx	245,754	23.263	1.665	16.908	26.306	Industry (2-digit) × Time

**Table 2:** Descriptive statistics of variables: Import.

Variable type	Name	N	Mean	Std	Min	Max^a^	Level
Outcome variables	LnIm	222,888	10.907	6.514	0	23.683	Province × Product × Time
	ImDensity	16,971	10.151	16.752	1	125	Province × Industry (2-digit) × Time
	VarIM	92,597	1.81509	6.038	0.001	77.182	Province × Product × Time
Explanatory variable	DTreat	222,888	0.067	0.251	0	1	Province × Time
Control variables	LnGdp	222,888	9.686	1.114	6.734	11.818	Province × Time
	LnWEx	222,888	24.94636	1.429	18.034	27.471	Industry (2-digit)× Time
	LnCIm	222,888	22.499	1.900	14.694	25.945	Industry (2-digit) × Time

#### 3.1.1. Control group.

The control group data was sourced from the five coastal regions closest to Hainan—Guangdong, Guangxi, Fujian, Shanghai, and Zhejiang. Although there are differences among these provinces, they generally exhibit highly similar import and export trends for comparable categories of products. Since 2015, these provinces have not undergone significant regional economic reforms, and their range of import and export products is extensive enough to cover 99.9% of the main import and export products of Hainan Province. Therefore, the control group was composed of the same import and export product codes as those used in Hainan Province. To further validate the effectiveness of the control group, parallel trend tests will be conducted in subsequent chapters.

This study employs a difference-in-differences (DID) strategy, for which the Stable Unit Treatment Value Assumption (SUTVA) is crucial. The SUTVA requires that the treatment applied in one region (Hainan) does not affect the outcomes in control regions. To ensure this assumption holds, we select control provinces whose economic scale is substantially greater than that of Hainan, making it highly unlikely that policy changes in Hainan could produce meaningful spillover effects on these provinces. Additionally, by restricting our analysis to products commonly traded by both Hainan and the control provinces, we further minimize the risk of trade diversion or creation effects arising from uniquely regional products. We also conduct parallel trend tests and placebo timing tests, both of which support the absence of significant spillover effects, thereby validating our identification strategy and ensuring the robustness of our DID estimates.

#### 3.1.2. Outcome variables.

***LnEx/ LnIm***: These two variables measure the trade level for each category of products with China’s major importing and exporting countries. All quarterly import and export data are sourced from the 6-digit HS codes provided by the “General Administration of Customs of the People’s Republic of China.” This study covers the period from the first quarter of 2015 to the first quarter of 2024. In empirical analysis, to retain data for zero trade flows, we increment the trade volume data by one and then convert it into natural logarithms. All logarithmic variables are processed in this manner.

***ExDensity/ lmDensity***: ExDensity and InDensity measure the degree of export and import diversification at the industry level. Specifically, for each province and each quarter, we first group products according to their 2-digit HS codes, which represent broad industry categories. Within each 2-digit HS category, we count the number of distinct 6-digit HS products that recorded non-zero trade volumes. This count reflects the variety of traded products within that industry and serves as an indicator of diversification in exports (ExDensity) and imports (InDensity).

**Fig 1:**
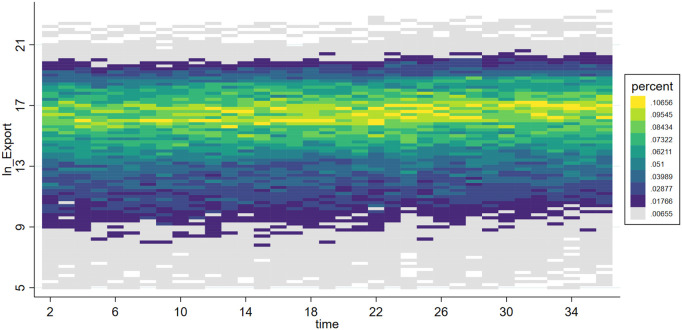
Scatter density plot of LnExport.

**Fig 2:**
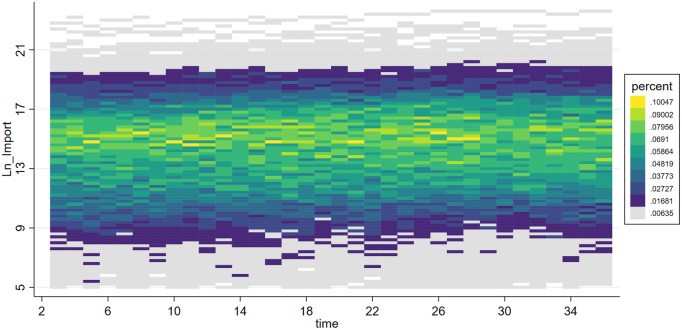
Scatter density plot of LnImport.

**Fig 3:**
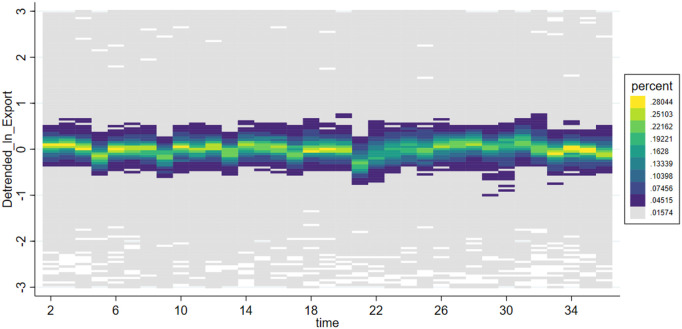
Scatter density plot of Detrended_LnExport.

**Fig 4:**
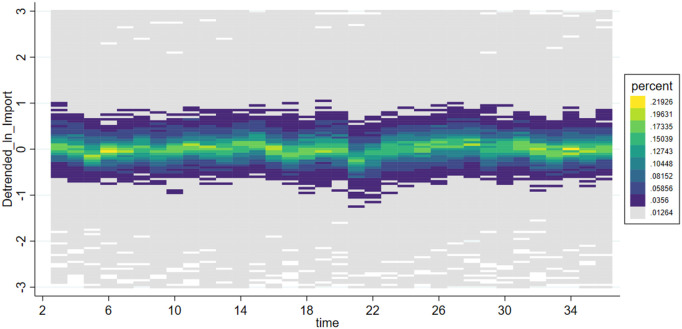
Scatter density plot of Detrended_LnImport.


***VarEx/ VarIm*** These two variables measure the volatility of imports and exports for each subcategory of products. Due to significant differences in supply cycles among different types of products, some have sparse quarterly import and export data. Therefore, we selected 6-digit HS coded products with non-zero trade volumes over more than 20 quarters as our analysis samples. This includes products ranked within the top 518 for import frequency and top 484 for export frequency. To analyze the volatility trends of these products, we first perform a second-order fitting to detrend the data, retaining only the volatility trends, resulting in detrended_Ex and detrended_Im. The effects before and after detrending are shown in Figs 1–4. We then calculate VarEx and VarIm using the detrended data along with the variance of the data from the preceding and following quarters. The specific formulas are presented as Equation 1 and Equation 2, where Var denotes the calculation of variance, t represents the current quarter, and t-1 and t + 1 represent the previous and next quarters, respectively.

$${VarEx}_{t}=Var(detrended\_{Ex}_{t-1}+detrended\_{Ex}_{t}\;+detrended\_{Ex}_{t+1})$$ (1)

$${VarIm}_{t}=Var(detrended\_{Im}_{t-1}+detrended\_{Im}_{t}\;+detrended\_{Im}_{t+1})$$ (2)

#### 3.1.3. Explanatory variables.

***DTreat:*** DTreat is derived from the product of the interaction term between D and Treat, which indicates whether a product has been affected by the HFTP policy. D is a dummy variable that takes the value of 0 or 1, representing whether the province is Hainan (1 for Hainan, 0 for others). Treat is also a dummy variable; considering that the HFTP policy was initiated in June 2020, we set Treat to 1 for the third quarter of 2020 and subsequent periods, and 0 for earlier periods. DTreat is set to 1 if the observation is from Hainan Province and the quarter is 2020Q3 or later; otherwise, DTreat is set to 0.

#### 3.1.4. Control variables.

***lnGdp*:** This variable uses the GDP of various regions to measure the impact of economic factors on trade. The data is sourced from China’s National Bureau of Statistics.

***lnWIm/lnWEx*:** These two variables further control for the intrinsic differences among various products. Since import and export trade is a global activity, changes in global demand for a specific category of products will affect China’s imports and exports of these products. lnWIm and lnWEx are based on the 2-digit HS codes corresponding to the 6-digit HS codes for industry categories, calculating the total imports and exports of these categories by major global trading nations, thus reflecting changes in international supply and demand for these industries. The data is sourced from UN Comtrade.

**lnCIm/lnCEx:** lnCIm and lnCEx capture the influence of national-level market conditions on regional trade outcomes. Specifically, for each industry category defined by its 2-digit HS code, we calculate the total value of China’s imports (CIm) and exports (CEx) to and from the global market. These values represent China’s total trade with the rest of the world for each industry and reflect international supply and demand dynamics. We then take the natural logarithm of these aggregate trade values to obtain lnCIm and lnCEx. The data are sourced from the General Administration of Customs of the People’s Republic of China.

### 3.2. Econometric model

To determine the causal relationship between the construction of the HFTP and product import and export, this study employed quasi-experimental models including Difference in Differences (DID) and Regression Discontinuity Design – Difference in Differences (RD-DD). The DID model has been widely used to analyze the impact of policies [39,40], and this study evaluates the “Overall Plan for the Construction of the HFTP” announced in June 2020 as an exogenous policy shock. Based on the explanatory variable DTreat, we classified the import and export products into control and treatment groups to compare the subgroup differences in export volumes before and after the policy impact. The baseline model for the DID design can be expressed by the following equation 3.


$$Y_{pct}=\beta_{0}+\beta_{1}{\text{ DTreat }}_{pct}+\sum {\mathrm{\backslash nolimits}}_{k=1}^{K}\;\gamma_{k}{\text{ Control }}_{pct}+\mu_{c}\;{\;{+\alpha }_{p}+{\text{year}}_{t}+\text{season }}_{t}+\epsilon_{pct}$$ (3)

In this equation, *p* represents the province, including six provinces; *c* represents the product, covering six-digit or two-digit HS codes; *t* represents the quarterly period, from the first quarter of 2015 to the first quarter of 2024. *Y*_*pct*_ denotes the outcome variables, namely the volume, category number, and volatility of imports and exports. *Control*_pct_ represents the respective control variables. $\mu_{c}$, $\alpha_{p}$, ${year}_{t}$ and ${season}_{t}$ respectively denote fixed effects for product, province, year, and season, while ɛ_*pct*_ represents the error term.

To validate Hypotheses 1 and 2 and explore the impact of the construction of the HFTP on product imports and exports, we employed a DID model as shown in equation 4 and equation 5. Here, lnEx and lnIm represent the export and import volumes, respectively, for a 6-digit product code in province *p* during period *t*. Exports are influenced by international demand for the product and national export levels; imports are influenced by international supply of the product and national import levels. Consequently, the control variables differ slightly for these two scenarios.


$$\begin{array}{c} {LnEx}_{pct}=\beta_{0}+\beta_{1}{\text{ DTreat }}_{pct}+\beta_{3}{LnGdp}_{pct}+{\beta_{4}LnWIm}_{pct}{{{\;+\;\beta_{5}LnCEx}_{pct}+\mu }_{c}\;{+\;\alpha }_{p}+{\text{year }}_{t}+\text{season }}_{t}+\epsilon_{pct} \end{array}$$ (4)

$$\begin{array}{c} {LnIm}_{pct}=\beta_{0}+\beta_{1}{\text{ DTreat }}_{pct}+{\beta_{3}LnGdp}_{pct}+{\beta_{4}LnWEx}_{pct}{{\;{{+\;\beta }_{5}LnCIm}_{pct}+\mu }_{c}\;{+\;\alpha }_{p}+{\text{year }}_{t}+\text{season }}_{t}+\epsilon_{pct} \end{array}$$ (5)

Similarly, to validate Hypotheses 3 and 4 and explore the impact of free trade port construction on the number of import and export categories, we employed the DID models as shown in equations 6 and 7. Here, ExDensity/ lmDensity represents the number of product categories corresponding to the 2-digit product codes in province p during period t. The control variables and fixed effects are the same as those in the previous model.


$$\begin{array}{c} {ExDensity}_{pct}=\beta_{0}+\beta_{1}{\text{ DTreat }}_{pct}+\sum {\mathrm{\backslash nolimits}}_{k=1}^{K}\;\gamma_{k}{\text{ Control }}_{pct}{{\;+\;\mu }_{c}\;{+\;\alpha }_{p}+{\text{year }}_{t}+\text{season }}_{t}+\epsilon_{pct} \end{array}$$ (6)

$$\begin{array}{c} {ImDensity}_{pct}=\beta_{0}+\beta_{1}{\text{ DTreat }}_{pct}+\sum {\mathrm{\backslash nolimits}}_{k=1}^{K}\;\gamma_{k}{\text{ Control }}_{pct}{{\;+\;\mu }_{c}\;{+\;\alpha }_{p}+{\text{year }}_{t}+\text{season }}_{t}+\epsilon_{pct} \end{array}$$ (7)

Likewise, to validate Hypotheses 5 and 6 and analyze the impact of free trade port construction on the volatility of imports and exports, we use VarExp and VarImp to measure the volatility during this period. The specific DID models are presented in equations 8 and 9, with control variables and fixed effects consistent with the earlier models.


$$\begin{array}{c} {VarEx}_{pct}=\beta_{0}+\beta_{1}{\text{ DTreat }}_{pct}+\sum {\mathrm{\backslash nolimits}}_{k=1}^{K}\;\gamma_{k}{\text{ Control }}_{pct}{{\;+\;\mu }_{c}\;{+\;\alpha }_{p}+{\text{year}}_{\;t}+\text{season }}_{t}+\epsilon_{pct} \end{array}$$ (8)

$$\begin{array}{c} {VarIm}_{pct}=\beta_{0}+\beta_{1}{\text{ DTreat }}_{pct}+\sum {\mathrm{\backslash nolimits}}_{k=1}^{K}\;\gamma_{k}{\text{ Control }}_{pct}{{\;+\;\mu }_{c}\;{+\;\alpha }_{p}+{\text{year }}_{t}+\text{season }}_{t}+\epsilon_{pct} \end{array}$$ (9)

Due to its unique design, Regression Discontinuity (RD) is considered more reliable than traditional “natural experiment” strategies such as Difference in Differences (DID) or instrumental variables [41], and has been widely utilized in numerous studies [42–44]. Particularly in fields like environmental and energy economics, recent empirical studies have adapted the RD framework to scenarios where time serves as a running variable and treatments begin at specific time thresholds [45]. Given the broad time range of our data, we employed the DID method to assess the long-term effects of the HFTP construction. To verify the short-term abrupt changes we are interested in, we referred to the Regression Discontinuity – Difference in Differences (RD-DD) regression method by Avdic and Karimi [40], using the RD-DD model in equations 10 and 11. This model exploits differences along the dimensions of products and time to detect jumps in covariates other than the core explanatory variable around a specific time threshold. It requires that no discontinuous changes in various covariates occur within the time window around the third quarter of 2020. The specific model follows, where f(d – c) is a local polynomial function of the forcing variable, employing a quadratic polynomial that allows different shapes on either side of c. The regression coefficient of interest is β2, representing the interaction between DTreat and f(d – c). This represents two types of differences: the first is the short-term difference before and after the construction of the HFTP, and the second is the difference between the treatment and control groups.

While the implementation of the Hainan Free Trade Port (HFTP) was uncertain for an extended period, it was not until June 2020 that the central government officially released the “Master Plan for the Construction of the Hainan Free Trade Port.” Before this announcement, no major trade liberalization measures—such as broad tariff exemptions, investment facilitation reforms, or customs streamlining policies—had been implemented or confirmed. Without concrete policy certainty, market participants had limited ability to anticipate the timing or scope of the changes. Therefore, although the treatment timing was determined by the government, it can be regarded as quasi-exogenous. To capture the policy’s short-term dynamic effects, we adopt a Regression Discontinuity in Time - Difference-in-Differences (RD-DD) framework, which accounts for both the discontinuous change at the threshold and differences between treated and control groups.


$$\begin{array}{c} {LnEx}_{pct}=\beta_{0}+{\beta_{1}{\text{ DTreat }}_{pct}+\beta }_{2}{\text{ DTreat }}_{pct}\times f(t-c)\\ +\;f\;(t-c)\sum {\mathrm{\backslash nolimits}}_{k=1}^{K}\;\gamma_{k}{\text{ Control }}_{pct}\;{+\mu }_{c}\;{+\;\alpha }_{p}+{\text{ year}}_{t}\\ {+\text{ season}}_{t}+\epsilon_{pct} \end{array}$$ (10)

$$\begin{array}{c} {LnIm}_{pct}=\beta_{0}+{\beta_{1}{\text{ DTreat }}_{pct}+\beta }_{2}{\text{ DTreat }}_{pct}\times f(t-c)\\ +\;f\;(t-c)\sum {\mathrm{\backslash nolimits}}_{k=1}^{K}\;\gamma_{k}{\text{ Control }}_{pct}\;{+\mu }_{c}\;{+\;\alpha }_{p}+{\text{ year}}_{t}\\ {+\text{ season}}_{t}+\epsilon_{pct} \end{array}$$ (11)

## 4. Empirical results

### 4.1. Necessity test

#### 4.1.1. Parallel trend test.

Both the Difference in Differences (DID) model and the Regression Discontinuity – Difference in Differences (RD-DD) model require a parallel trends test to ensure unbiased results. This means that before the implementation of the HFTP policy, the outcome variable trends for both the experimental and control groups must be similar; however, these trends may diverge after the policy implementation. Equation (12) specifies the event study model used to test the parallel trends assumption. In this model, Ypct represents the outcome variable, including the logarithm of export or import volumes, product diversity, or trade volatility. The terms Prek, Currentpct, and Postk are sets of dummy variables indicating, respectively, the k-th quarter before the policy implementation, the quarter of the policy implementation (2020Q3), and the k-th quarter after the policy. The quarter immediately preceding the policy implementation (2020Q2) is omitted as the baseline period.

**Fig 5:**
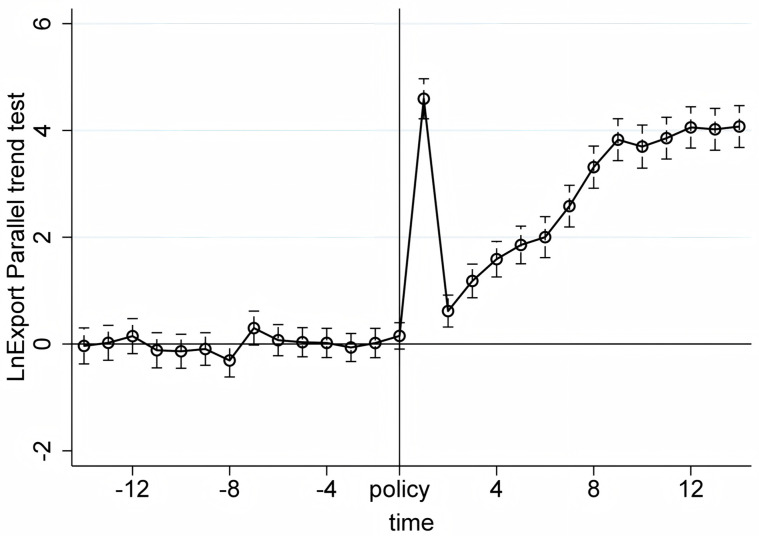
Parallel trend test of LnExport.

**Fig 6:**
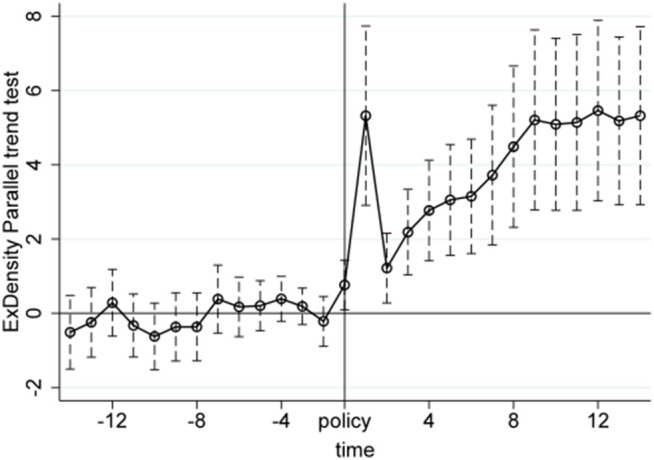
Parallel trend test of ExDensity.

**Fig 7:**
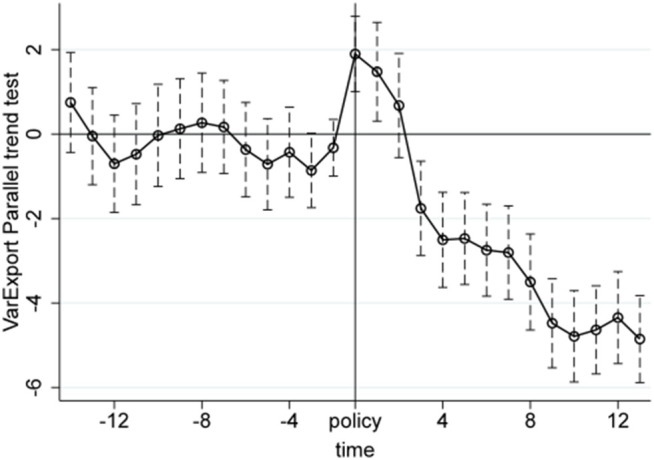
Parallel trend test of VarExport.

**Fig 8:**
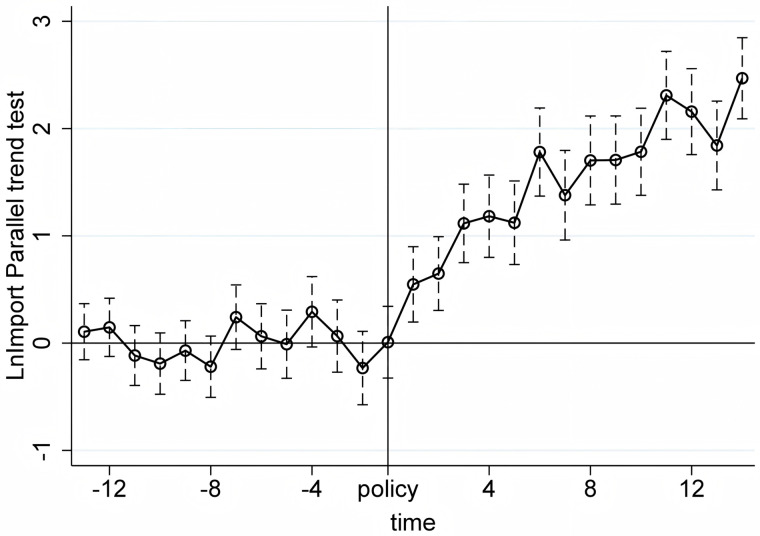
Parallel trend test of LnImport.

**Fig 9:**
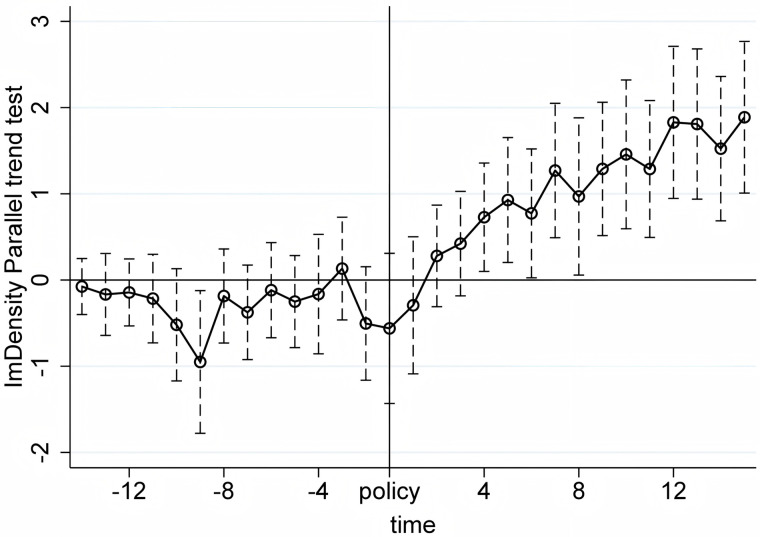
Parallel trend test of ImDensity.

**Fig 10:**
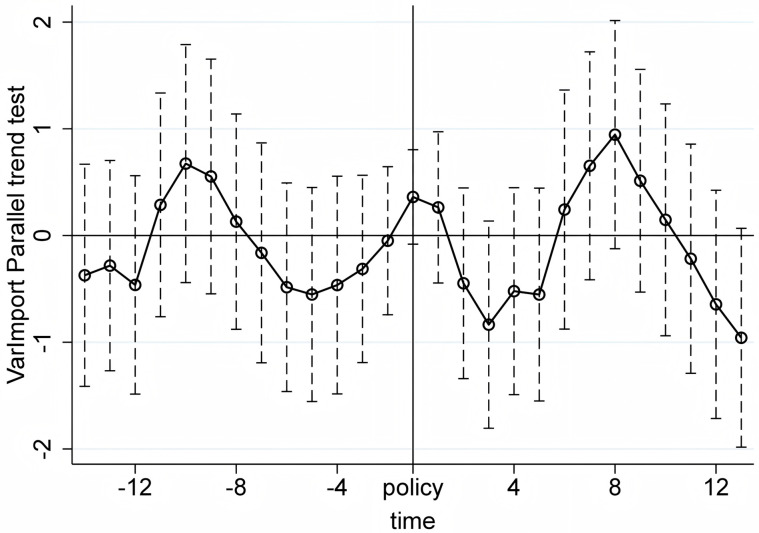
Parallel trend test of VarImport.


We conduct the parallel trend tests using quarterly product-level import and export data from the first quarter of 2017 to the first quarter of 2024. The X-axis in Figs 5–10 represents event time, where t = 0 corresponds to the policy announcement quarter (2020Q3), and each tick indicates the number of quarters before or after that quarter (e.g., t = −1 is 2020Q2, t = 1 is 2020Q4). The Y-axis shows the coefficient estimates of the interaction between treatment status and time, derived from an event study specification. These coefficients represent the difference in outcomes between the treatment and control groups in each quarter relative to the baseline quarter t = −1 (2020Q2), which is omitted from the regression and serves as the reference point. The results, displayed under a 95% confidence interval in Figs 5–10, indicate that before the policy implementation, the experimental and control groups maintained parallel trends. After the policy enactment, the groups exhibited varying degrees of non-parallel trends. This supports the parallel trends assumption of our DID and RD-DD analysis. Additionally, we observe that export products experienced a significant short-term deviation immediately after the policy implementation, followed by a rapid return to the pre-existing trend in the subsequent quarter.

$$\begin{array}{c} Y_{pct}=\beta_{0}+\sum_{k=-K}^{-1}\;\delta_{k}{\text{Pre}}_{k,pct}+\delta_{0}{\text{Current}}_{pct}+\sum_{k=1}^{K}\;\delta_{k}{\text{Post}}_{k,pct}\\ \;+\sum_{m=1}^{n}\;\gamma_{m}{\text{Control}}_{pct}{+\mu }_{c}{+\alpha }_{p}+\;{\text{year}}_{t}{+\text{season}}_{t}+\epsilon_{pct} \end{array}$$ (12)

#### 4.1.2. Covariate continuity test.

**Fig 11:**
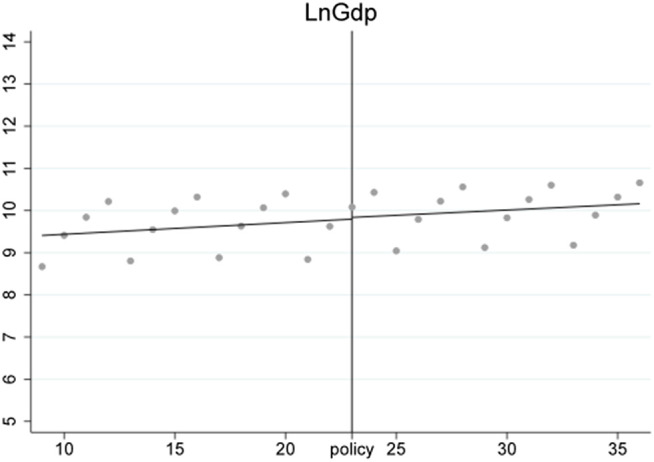
Changes of covariate LnGdp.

**Fig 12:**
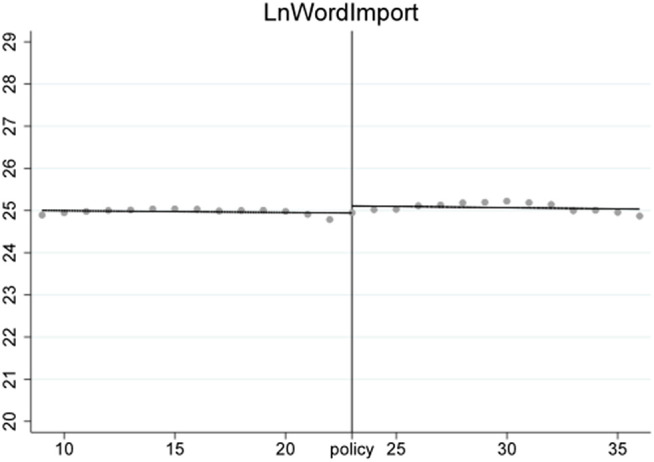
Changes of covariate LnWIm.

**Fig 13:**
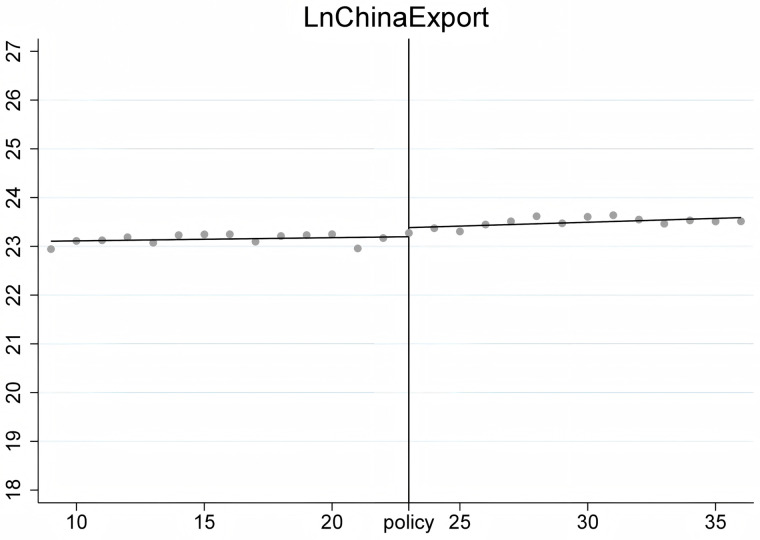
Changes of covariate LnCEx.

**Fig 14:**
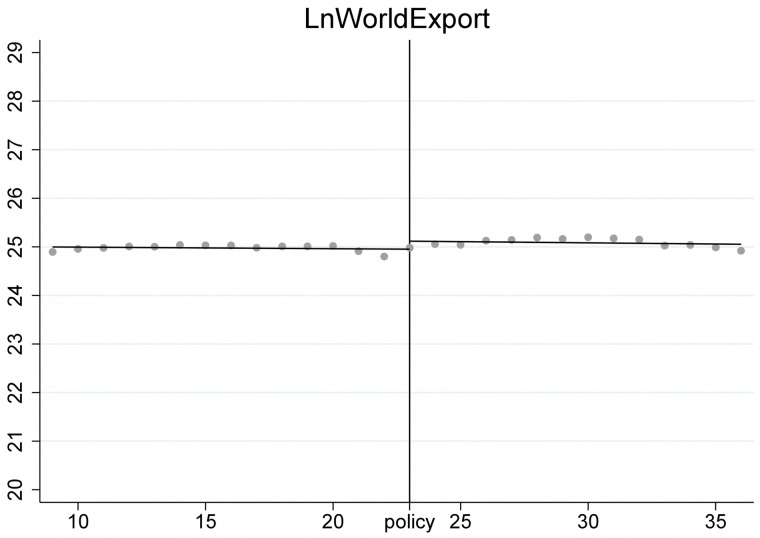
Changes of covariate LnWEx.

**Fig 15:**
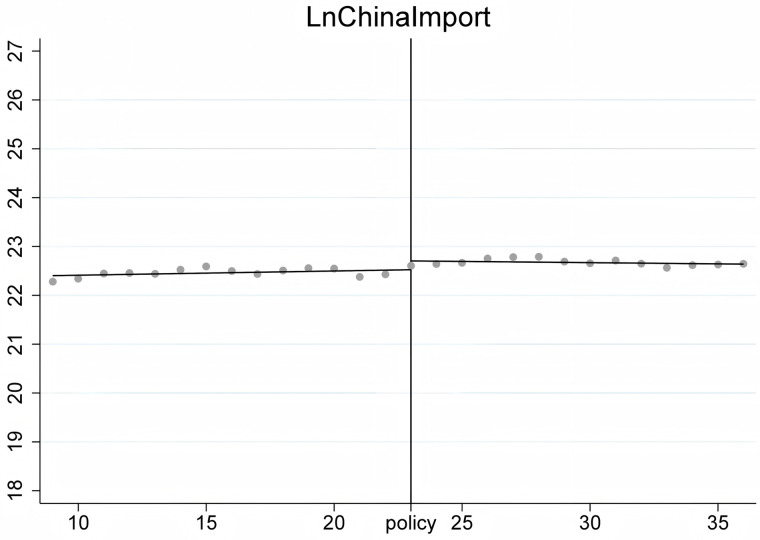
Changes of covariate LnCIm.

To ensure the stability of the Regression Discontinuity – Difference in Differences (RD-DD) results, it is crucial that the covariates do not undergo abrupt changes. Therefore, we conducted an RD-DD regression analysis treating all covariates as dependent variables. Figs 11–15 display the estimation results for key covariates. In these plots, the X-axis represents actual calendar time measured in quarters, with the policy announcement quarter marked by the vertical line. The Y-axis shows the level of the respective covariate, such as LnWorldImport in Fig 11. No significant discontinuity is observed in any covariate immediately before or after the policy, suggesting that there are no abrupt structural breaks at the threshold. This supports the assumption that the assignment of treatment is as good as random around the cutoff, thus satisfying the identifying assumptions of the RD-DD framework.

### 4.2. DID results

We conducted regression analyses using Equations 3–9, and the results for imports and exports are presented in Tables 3 and 4 respectively. Initially, columns 1, 4, and 7, which do not include control variables, showed that the significance of outcomes is not influenced by the inclusion of control variables. The results in columns 2, 5, and 8 cover the period from the first quarter of 2015 to the first quarter of 2024. We consider the outcomes in columns 3, 6, and 9 as our final DID analysis results. In terms of exports, there was a significant increase in export value, notable enhancement in the diversification of export products, and a significant reduction in export volatility, thus validating Hypotheses 1, 3, and 5. On the import side, although there was a significant increase in both the value and diversification of imports, the volatility of imports showed no significant changes, possibly due to the sensitivity of import volatility to global supply and demand fluctuations, thereby validating Hypotheses 2 and 4, but not Hypothesis 6. Moreover, the data reveals that the coefficients for the explanatory variables for imports are lower than those for exports, indicating that the HFTP has a greater positive impact on exports than on imports. This asymmetry may be attributed to several factors. In the early stages of the HFTP’s implementation, policy incentives and institutional support were more directly oriented toward boosting export-oriented industries, while the full liberalization of imports, particularly consumer goods and intermediate inputs, requires a longer adjustment period. Additionally, differences in international market access, product structures, and the initial logistical infrastructure may have contributed to the faster and stronger export response observed in the data.

**Table 3:** Difference-in-differences regression results for export.

	(1)	(2)	(3)	(4)	(5)	(6)	(7)	(8)	(9)
Time(year.season)	2015.1-2024.1	2015.1-2024.1	2017.1-2024.1	2015.1-2024.1	2015.1-2024.1	2017.1-2024.1	2015.1-2024.1	2015.1-2024.1	2017.1-2024.1
Variable	${LnEx}_{pct}$	${LnEx}_{pct}$	${LnEx}_{pct}$	ExDensity	ExDensity	ExDensity	${VarEx}_{pct}$	${VarEx}_{pct}$	${VarEx}_{pct}$
TreatD	2.566^***^	2.506^***^	2.621^***^	3.832^***^	3.807^***^	3.830^***^	−2.292^***^	−2.200^***^	−1.970^***^
	(0.114)	(0.114)	(0.111)	(0.998)	(0.998)	(0.947)	(0.295)	(0.296)	(0.306)
Control variables	NO	Yes	Yes	NO	Yes	Yes	NO	Yes	Yes
Constant	Yes	Yes	Yes	Yes	Yes	Yes	Yes	Yes	Yes
Product fixed effect	Yes	Yes	Yes	Yes	Yes	Yes	Yes	Yes	Yes
Province fixed effect	Yes	Yes	Yes	Yes	Yes	Yes	Yes	Yes	Yes
year fixed effect	Yes	Yes	Yes	Yes	Yes	Yes	Yes	Yes	Yes
season fixed effect	Yes	Yes	Yes	Yes	Yes	Yes	Yes	Yes	Yes
N	245,754	245,754	192,618	17,044	17,044	13,505	104,008	104,008	84,019
R^2^	0.603	0.604	0.608	0.952	0.963	0.959	0.202	0.202	0.193

Notes: *, **, *** indicate significance at the 10%, 5%, and 1% levels, respectively; the values in parentheses of the regression coefficients are the standard errors clustered at the product level; the same notes apply to the tables below

**Table 4:** Difference-in-differences regression results for import.

	(1)	(2)	(3)	(4)	(5)	(6)	(7)	(8)	(9)
Time(year.season)	2015.1-2024.1	2015.1-2024.1	2017.1-2024.1	2015.1-2024.1	2015.1-2024.1	2017.1-2024.1	2015.1-2024.1	2015.1-2024.1	2017.1-2024.1
Variable	${LnIm}_{pct}$	${LnIm}_{pct}$	${LnIm}_{pct}$	ImDensity	ImDensity	ImDensity	${VarIm}_{pct}$	${VarIm}_{pct}$	${VarIm}_{pct}$
TreatD	1.471^***^	1.426^***^	1.366^***^	1.376^***^	1.378^***^	1.285^***^	−0.307	−0.312	− 0.059
	(0.119)	(0.118)	(0.115)	(0.292)	(0.292)	(0.275)	(0.278)	(0.268)	(0.276)
Control variables	NO	Yes	Yes	NO	Yes	Yes	NO	Yes	Yes
Constant	Yes	Yes	Yes	Yes	Yes	Yes	Yes	Yes	Yes
Product fixed effect	Yes	Yes	Yes	Yes	Yes	Yes	Yes	Yes	Yes
Province fixed effect	Yes	Yes	Yes	Yes	Yes	Yes	Yes	Yes	Yes
year fixed effect	Yes	Yes	Yes	Yes	Yes	Yes	Yes	Yes	Yes
season fixed effect	Yes	Yes	Yes	Yes	Yes	Yes	Yes	Yes	Yes
N	222,888	222,888	174,696	16,971	16,971	13,369	92,597	92,597	76,786
R^2^	0.571	0.572	0.576	0.949	0.949	0.952	0.184	0.184	0.184

To further interpret the magnitude of the effects, we refer to the specific coefficient estimates. In Table 3, Column (2) and Table 4, Column (2), the estimated coefficients for the treatment effect are 2.506 and 1.426 respectively, suggesting that the HFTP had a substantial positive impact on the average trade value per product. This finding is broadly consistent with the growth trend reported by the General Administration of Customs of China, which shows that Hainan’s total trade volume increased by approximately 260% and export volumes by about 384% from 2020 to 2024. While the exact magnitudes differ, the direction of strong trade growth is similar. In contrast, other coastal provinces experienced much slower trade growth during the same period, especially under the impact of the COVID-19 pandemic.

Similarly, the results in Table 3, Column (5) and Table 4, Column (5) indicate that the construction of the HFTP led to an average increase of 3.807 and 1.378 six-digit HS code product categories per quarter within each two-digit HS sector for exports and imports, respectively, thereby significantly promoting product diversification in Hainan Province.

Regarding trade volatility, Table 3, Column (8) and Table 4, Column (8) show that the variance of export values per product within each six-digit HS code sector decreased significantly by approximately 2.200 units per quarter following the implementation of the HFTP. In contrast, the changes in the variance of import values were not statistically significant. This suggests that the HFTP contributed to enhancing the stability of export trade volumes, while its impact on the volatility of import trade volumes remained limited.

### 4.3. RD-DD results

We assessed the long-term effects of the construction of the HFTP using the DID model and examined the short-term effects through RD-DD regression analyses via Equations 10 and 11, as presented in Table 5. To determine if there were sudden changes at the breakpoints, we narrowed the timeframe to one year. Columns 1 and 4 use the two quarters before the implementation of the HFTP as breakpoints, columns 2 and 5 use the quarter of implementation, and columns 3 and 6 use the two quarters after implementation. The results for exports indicate no significant changes prior to the policy, but a notable surge immediately after the policy announcement, followed by a gradual stabilization. For imports, no significant short-term discontinuities are detected around the policy implementation period.

**Table 5:** RD-DD results.

	(1)	(2)	(3)	(4)	(5)	(6)
Time(year.season)	2019.3-2020.3	2020.1-2021.1	2020.3-2021.3	2019.3-2020.3	2020.1-2021.1	2020.3-2021.3
Breakpoint	2020.1	2020.3	2021.1	2020.1	2020.3	2021.1
Variable	${LnEx}_{pct}$	${LnEx}_{pct}$	${LnEx}_{pct}$	${LnIm}_{pct}$	${LnIm}_{pct}$	${LnIm}_{pct}$
TreatD	0.029	6.204^***^	−1.729^***^	0.098	0.821	0.174
	(0.153)	(0.222)	(0.170)	(0.196)	(0.897)	(0.142)
Control variables	Yes	Yes	Yes	Yes	Yes	Yes
Product fixed effect	Yes	Yes	Yes	Yes	Yes	Yes
Province fixed effect	Yes	Yes	Yes	Yes	Yes	Yes
year fixed effect	Yes	Yes	Yes	Yes	Yes	Yes
season fixed effect	Yes	Yes	Yes	Yes	Yes	Yes
N	26,568	26,568	26,568	24,096	24,096	24,096
R^2^	0.705	0.703	0.688	0.635	0.631	0.627

**Fig 16:**
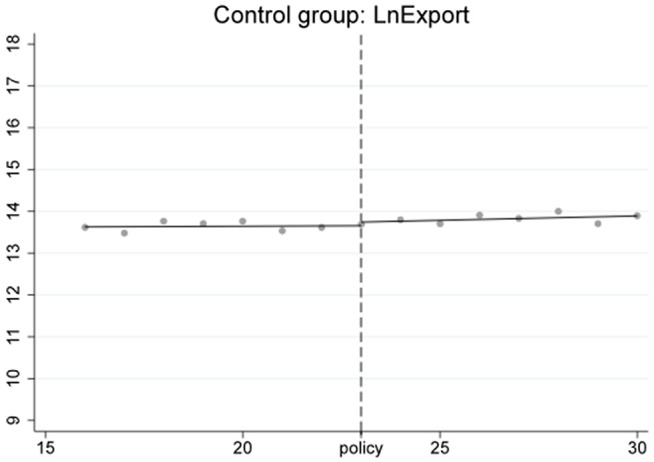
RD result of control group.

**Fig 17:**
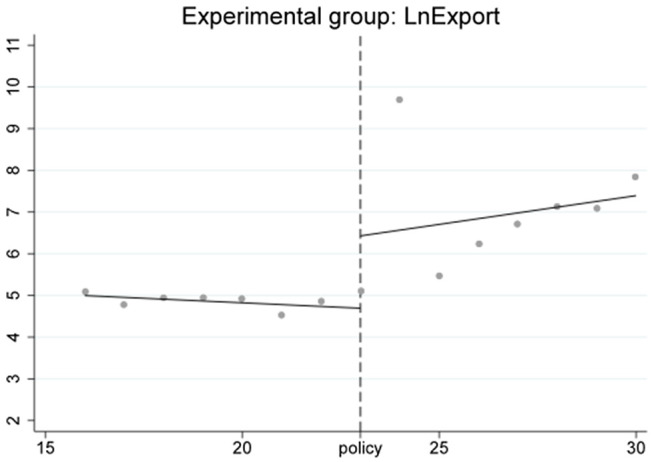
RD result of experimental group.

**Fig 18:**
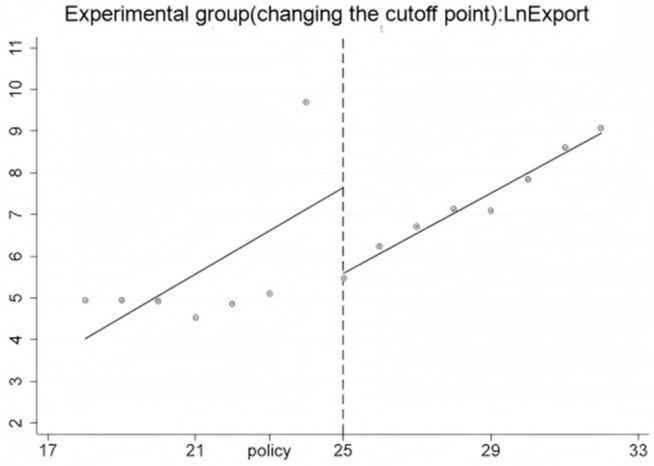
RD result of experimental group after changing the cutoff point.

The RD-DD results focus solely on capturing short-term fluctuations immediately surrounding the policy implementation. The observed jump in export volumes in the first quarter after the HFTP announcement is consistent with a short-term surge effect, while the absence of abrupt changes in imports suggests limited immediate impact.Figs 16–18 further illustrate these short-term dynamics. In particular, Figs 17 and 18 show notable outliers in the first quarter after the policy implementation (2020Q4), corresponding to a surge in export activities. This pattern is expected, as major policy announcements often trigger concentrated trading efforts to capitalize on early opportunities, leading to short-term spikes. After this initial surge, export volumes return to a more stable trajectory, indicating that the broader effects of the HFTP will continue to evolve over time.

## 5. Robustness tests

### 5.1. Counterfactual test for replacing sample

In order to further substantiate the reliability of our findings, we used data from Hainan Province as the control group and data from the original control group as the experimental group for a counterfactual analysis. If the regression results of the counterfactual sample align closely with the baseline regression results, it may indicate bias due to some unobserved factors. Conversely, if the counterfactual results are contrary to the baseline, it confirms the validity of our conclusions. As shown in Table 6, the test results for the swapped sample provinces indicate significant increases in both export and import volumes as well as diversification, significant rise in export volatility, and insignificant changes in import volatility. These findings support the reliability of the DID method results.

**Table 6:** Counterfactual test for replacing sample.

Variable	${LnEx}_{pct}$	${LnIm}_{pct}$	${ExDensity}_{pct}$	${ImDensity}_{pct}$	${VarEx}_{pct}$	${VarIm}_{pct}$
TreatD	−1.663^***^	−0.878^***^	−2.238^***^	− 0.730^***^	1.530^***^	−0.037
	(0.073)	(0.077)	(0.545)	(0.170)	(0.185)	(0.178)
Control variables	Yes	Yes	Yes	Yes	Yes	Yes
Constant	Yes	Yes	Yes	Yes	Yes	Yes
FE	Yes	Yes	Yes	Yes	Yes	Yes
N	192,618	174,696	13,505	13,369	84,019	76,786
R^2^	0.606	0.576	0.959	0.952	0.194	0.184

### 5.2. Counterfactual test for replacing policy implementation time

To further confirm the reliability of our findings, we artificially constructed the implementation timeline for the HFTP, setting a counterfactual policy date in the third quarter of 2017. We then validated this within the timeframe from the first quarter of 2015 to the first quarter of 2020. The regression results, shown in Table 7, indicate that except for a slight decrease in export volumes and import volatility, other results were not significant, further affirming the reliability of our findings.

**Table 7:** Counterfactual test for replacing policy implementation time.

Variable		${LnEx}_{pct}$	${LnIm}_{pct}$		${ExDensity}_{pct}$	${ImDensity}_{pct}$	${VarEx}_{pct}$	${VarIm}_{pct}$
TreatD	−0.621^***^		−0.152	−0.232		− 0.034	−0.538	−0.738^**^
	(0.092)		(0.093)	(0.258)		(0.133)	(0.339)	(0.270)
Control variables	Yes		Yes	Yes		Yes	Yes	Yes
Constant	Yes		Yes	Yes		Yes	Yes	Yes
FE	Yes		Yes	Yes		Yes	Yes	Yes
N	139,482		126,504	9,629		9,568	59,052	51,585
R^2^	0.676		0.630	0.935		0.944	0.248	0.186

### 5.3. Bandwidth sensitivity test

The accuracy of parameter estimation in RD-DD heavily depends on the choice of bandwidth. A narrower bandwidth reduces estimation bias but may increase variance due to a smaller sample size, thus impacting the precision of the estimate. Conversely, a wider bandwidth increases sample capacity but might incorrectly reject the null hypothesis of “no local treatment effect”. Tables 8 present the results of the bandwidth sensitivity tests at two breakpoints for export volumes. The results are consistent with the previous regression conclusions, demonstrating the robustness of the RD-DD test outcomes.

**Table 8:** Bandwidth sensitivity test.

	(1)	(2)	(3)	(4)	(5)	(6)
Time(year.season)	2020.1-2021.1	2019.4-2021.2	2019.3-2022.3	2020.3-2021.3	2020.2-2021.4	2019.1-2022.1
Breakpoint	2020.3	2020.3	2020.3	2021.1	2021.1	2021.1
Bandwidth multiplier	1	1.5	2	1	1.5	2
Variable	${LnEx}_{pct}$	${LnEx}_{pct}$	${LnEx}_{pct}$	${LnEx}_{pct}$	${LnEx}_{pct}$	${LnIm}_{pct}$
TreatD	6.204^***^	13.560^***^	10.078^***^	−1.729^***^	−3.446^***^	−1.671^***^
	(0.222)	(0.55)	(0.375)	(0.170)	(0.443)	(0.311)
Control variables	Yes	Yes	Yes	Yes	Yes	Yes
FE	Yes	Yes	Yes	Yes	Yes	Yes
N	26,568	39,852	53,136	26,568	39,852	53,136
R^2^	0.703	0.704	0.703	0.686	0.686	0.647

### 5.4. Change the fitting polynomial test

The choice of the fitting polynomial also impacts the accuracy of parameter estimates in RD-DD. Opting for polynomials of lower order might introduce high bias as they fail to capture complex relationships in the data. Conversely, higher-order polynomials, while providing a better fit, increase the variance of estimates, potentially leading to overfitting issues. Tables 9 display the sensitivity test results for different polynomial orders at the breakpoint. These results are consistent with previous regression analyses, affirming the robustness of the RD-DD findings.

**Table 9:** Changing the fitting polynomial test.

	(1)	(2)	(3)	(4)	(5)	(6)	(7)	(8)	(9)	(10)
Time(year.season)	2020.1-2021.1	2020.1-2021.1	2020.1-2021.1	2020.1-2021.1	2020.1-2021.1	2020.3-2021.3	2020.3-2021.3	2020.3-2021.3	2020.3-2021.3	2020.3-2021.3
Breakpoint	2020.3	2020.3	2020.3	2020.3	2020.3	2021.1	2021.1	2021.1	2021.1	2021.1
Polynomial degree of fitting	1	3	4	5	6	1	3	4	5	6
Variable	${LnEx}_{pct}$	${LnEx}_{pct}$	${LnEx}_{pct}$	${LnEx}_{pct}$	${LnEx}_{pct}$	${LnEx}_{pct}$	${LnEx}_{pct}$	${LnEx}_{pct}$	${LnEx}_{pct}$	${LnEx}_{pct}$
TreatD	8.938^***^	5.423^***^	5.111^***^	4.970^***^	4.902^***^	−1.907^***^	−1.678^***^	−1.657^***^	−1.648^***^	−1.644^***^
	(0.324)	(0.196)	(0.187)	(0.183)	(0.181)	(0.240)	(0.154)	(0.149)	(0.147)	(0.146)
Control variables	Yes	Yes	Yes	Yes	Yes	Yes	Yes	Yes	Yes	Yes
FE	Yes	Yes	Yes	Yes	Yes	Yes	Yes	Yes	Yes	Yes
N	26,568	26,568	26,568	26,568	26,568	26,568	26,568	26,568	26,568	26,568
R^2^	0.703	0.703	0.703	0.703	0.703	0.686	0.686	0.686	0.686	0.686

### 5.5. Robustness to alternative clustering levels

To assess the sensitivity of our results to the clustering level, we additionally cluster standard errors at the 2-digit HS product category level rather than the 6-digit product level. This accounts for potential unobserved shocks affecting broader industry categories. The results, presented in Table 10, remain consistent with our baseline findings, indicating robustness to alternative clustering structures.

**Table 10:** Changing Clustering Levels(2-digit HS).

Variable	${LnEx}_{pct}$	${LnIm}_{pct}$	${VarEx}_{pct}$	${VarIm}_{pct}$
TreatD	2.506^***^	1.426^***^	−2.240^***^	−0.314
	(0.323)	(0.227)	(0.682)	(0.359)
Control variables	Yes	Yes	Yes	Yes
Constant	Yes	Yes	Yes	Yes
FE	Yes	Yes	Yes	Yes
N	245754	222,888	104008	92579
R^2^	0.604	0.572	0.202	0.184

### 5.6. Robustness to alternative fixed effects specifications

We also test the robustness of our findings to stricter fixed effects specifications. Specifically, we include product-by-time fixed effects (product-quarter), which control for time-varying unobserved shocks at the product level. As shown in Table 11, the results remain robust, reinforcing the validity of our causal identification strategy.

**Table 11:** Changing fixed effects.

Variable	${LnEx}_{pct}$	${LnIm}_{pct}$	${ExDensity}_{pct}$	${ImDensity}_{pct}$	${VarEx}_{pct}$	${VarIm}_{pct}$
TreatD	2.405^***^	1.332^***^	2.270^***^	0.878^***^	−2.186^***^	−0.229
	(0.114)	(0.119)	(0.494)	(0.206)	(0.299)	(0.279)
Control variables	Yes	Yes	Yes	Yes	Yes	Yes
Constant	Yes	Yes	Yes	Yes	Yes	Yes
Product fixed effect	Yes	Yes	Yes	Yes	Yes	Yes
Province fixed effect	Yes	Yes	Yes	Yes	Yes	Yes
season fixed effect	Yes	Yes	Yes	Yes	Yes	Yes
Product×season fixed effect	Yes	Yes	Yes	Yes	Yes	Yes
N	245,754	222,888	16,194	16200	104,008	92597
R^2^	0.608	0.567	0.958	0.954	0.198	0.184

## 6. Heterogeneity analysis

The 2-digit HS codes classify all traded products into 98 categories. Based on these, all 6-digit HS codes are grouped into 10 major categories, covering detailed classifications of all global imports and exports as shown in Table 12. We conducted grouped Difference in Differences (DID) regression on the nine categories, excluding Category 10 (Special Transactions and Unclassified Products), using import and export volumes as outcome variables to observe the heterogeneous impact of HFTP construction on products from different industries. The results for imports and exports are shown in Tables 13 and 14, respectively.

**Table 12:** Trade product categories.

Category	Name	HS chapter	Category	Name	HS chapter
1	Agricultural Products and Food	1-24	6	Stone, Ceramics and Glass Products	68-70
2	Mineral Products and Chemical Products	25-38	7	Base Metals and Articles	72-83
3	Plastic and Rubber Products	39-40	8	Machinery, Equipment and Transportation Tools	84-89
4	Leather, Wood and Paper Products	41-49	9	Precision Instruments and Other Articles	90-97
5	Textiles and Clothing	50-63	10	Special Transactions and Unclassified Products	98

**Table 13:** Heterogeneity analysis: Export.

Category	(1)	(2)	(3)	(4)	(5)	(6)	(7)	(8)	(9)
Variable	${LnEx}_{pct}$	${LnEx}_{pct}$	${LnEx}_{pct}$	${LnEx}_{pct}$	${LnEx}_{pct}$	${LnEx}_{pct}$	${LnEx}_{pct}$	${LnEx}_{pct}$	${LnEx}_{pct}$
TreatD	1.127^*^	2.070^***^	2.986^***^	2.348^***^	1.388^***^	3.127^***^	3.064^***^	3.664^***^	1.822^***^
	(0.531)	(0.273)	(0.349)	(0.502)	(0.292)	(0.554)	(0.246)	(0.203)	(0.387)
Control variables	Yes	Yes	Yes	Yes	Yes	Yes	Yes	Yes	Yes
Constant	Yes	Yes	Yes	Yes	Yes	Yes	Yes	Yes	Yes
FE	Yes	Yes	Yes	Yes	Yes	Yes	Yes	Yes	Yes
N	13,224	31,494	16,182	8,700	18,792	7,656	28,710	51,852	15,660
R^2^	0.486	0.535	0.694	0.740	0.667	0.656	0.681	0.664	0.561

**Table 14:** Heterogeneity analysis: Import.

Category	(1)	(2)	(3)	(4)	(5)	(6)	(7)	(8)	(9)
Variable	${LnIm}_{pct}$	${LnIm}_{pct}$	${LnIm}_{pct}$	${LnIm}_{pct}$	${LnIm}_{pct}$	${LnIm}_{pct}$	${LnIm}_{pct}$	${LnIm}_{pct}$	${LnIm}_{pct}$
TreatD	2.639^*^	2.295^***^	2.683^***^	0.530	0.583^*^	1.718	0.402	0.525^***^	1.380^***^
	(0.341)	(0.346)	(0.662)	(0.451)	(0.233)	(0.925)	(0.359)	(0.180)	(0.387)
Control variables	Yes	Yes	Yes	Yes	Yes	Yes	Yes	Yes	Yes
Constant	Yes	Yes	Yes	Yes	Yes	Yes	Yes	Yes	Yes
FE	Yes	Yes	Yes	Yes	Yes	Yes	Yes	Yes	Yes
N	26,274	28,188	10,266	9,396	20,184	2,262	12,180	42,108	19,140
R^2^	0.552	0.562	0.697	0.583	0.586	0.589	0.587	0.632	0.564

From the export results in Table 13, it is evident that export volumes have significantly increased across all industries. However, the least increase in export volumes is seen in Category 1 (Agricultural Products and Food) and Category 5 (Textiles and Clothing), while high-tech products in Categories 7 and 8 (Base Metals and Articles, Machinery, Equipment, and Transportation Tools) have seen relatively higher growth. This indicates that the enhanced support for high-tech industries in Hainan has been effective.

Regarding imports, the growth in import volumes is more uneven across different categories. As shown in Table 14, Categories 1, 2, and 3 (Agricultural Products and Food, Mineral Products and Chemical Products, and Plastic and Rubber Products) experienced the most significant import growth. In contrast, high-tech products in Categories 7 and 8 saw less growth. The growth in imports for Categories 4, 6, and 7 (Leather, Wood and Paper Products, Stone, Ceramics and Glass Products, and Base Metals and Articles) was not significant.

The results of the heterogeneity analysis indicate that after the construction of the HFTP, the overall growth in export volumes exceeds that of import volumes. Additionally, the impact of the HFTP construction on export products is broader and deeper across different industries compared to import products.

## 7. Conclusions and policy implications

Determining how to further expand reforms and open up to enhance economic vitality is a critical concern for the Chinese government. The development of the HFTP marks the largest reform of economic special zones in China to date. This significant policy initiative is crucial not only for China’s efforts to expand globalization and deepen reforms but also provides valuable insights for other developing countries. Utilizing quarterly panel data of 2,111 trade products from six coastal provinces in China, from the first quarter of 2015 to the first quarter of 2024, we applied the Difference in Differences (DID) and Regression Discontinuity Design – Difference in Differences (RD-DD) models to empirically assess the impact of HFTP construction on import and export trade. The research findings are summarized as follows:

First, the HFTP policy significantly increased trade volumes, enhanced the diversification of import and export products, and reduced the volatility of export trade. However, the impacts on imports and exports are imbalanced, with a more pronounced positive effect on exports than on imports, and the volatility of import trade has not been significantly reduced. Additionally, a noticeable short-term surge in export volumes was observed immediately following the policy announcement. Moreover, from the perspective of product categories, the impact of HFTP construction is heterogeneous: it has lesser effects on agricultural exports and more significant effects on high-tech exports; for imports, it has greater effects on agricultural and textile products, but lesser effects on high-tech and metal products.

Based on our empirical findings and main conclusions, we propose the following recommendations:

First, it is essential to further refine and deepen the HFTP’s policy framework. While current initiatives such as zero-tariff programs, customs facilitation, and service liberalization have demonstrated positive effects, continued expansion of zero-tariff coverage, refinement of investment negative lists, and strengthening of legal and institutional frameworks are necessary to sustain momentum.

Second, balancing export-driven growth with the expansion of domestic demand is critical. Strengthening regional market integration and leveraging Hainan’s role as a consumption and logistics hub could help anchor stable import growth and broaden the economic base.

Third, trade risk management mechanisms should be enhanced. Developing early warning systems for external shocks, diversifying supply sources, and supporting enterprises in adapting to international uncertainty can help mitigate volatility risks.

Fourth, sectoral support policies should be aligned with long-term structural goals, prioritizing high-end manufacturing, digital economy, green energy, and modern services through targeted incentives, talent programs, and global innovation partnerships.

Finally, the HFTP experience offers valuable insights for other developing countries: institutional innovation, phased liberalization strategies, and coherent alignment between trade, investment, and industrial policies are key to building successful free trade ports. Sustainable success requires moving beyond tax incentives to establish comprehensive, transparent governance frameworks, robust infrastructure support, and sustained investment in human capital.

## Supporting information

S1 File S1 Table. HS codes of exported products. S2 Table. HS codes of imported products. S3 Table. RDD tests with polynomial fitting (bandwidth multiplier = 1.5). S4 Table. RDD tests with polynomial fitting (bandwidth multiplier = 2). S5 Table. Detailed parallel trend tests for LnExport. S6 Table. Detailed parallel trend tests for ExDensity. S7 Table. Detailed parallel trend tests for VarExport. S8 Table. Detailed parallel trend tests for LnImport. S9 Table. Detailed parallel trend tests for ImDensity. S10 Table. Detailed parallel trend tests for VarImport.
(ZIP)

S1 Appendix Major Trading Partners and Total Trade Volume (USD). (DOCX)
