# Supplementary material for: The impact of free trade port construction on regional import and export: Evidence from Hainan
Source: PLoS One. 2025 Aug 13;20(8):e0328875. doi: 10.1371/journal.pone.0328875 (PMC12349091; doi:10.1371/journal.pone.0328875)
Supplement: S1 File — S1 Table. HS codes of exported products. S2 Table. HS codes of imported products. S3 Table. RDD tests with polynomial fitting (bandwidth multiplier = 1.5). S4 Table. RDD tests with polynomial fitting (bandwidth multiplier = 2). S5 Table. Detailed parallel trend tests for LnExport. S6 Table. Detailed parallel trend tests for ExDensity. S7 Table. Detailed parallel trend tests for VarExport. S8 Table. Detailed parallel trend tests for LnImport. S9 Table. Detailed parallel trend tests for ImDensity. S10 Table. Detailed parallel trend tests for VarImport. (ZIP) [file pone.0328875.s001.zip › S1 Table. HS codes of exported products.docx]

**S1 Table. HS codes of exported products.**

Export:

| 030323,030354,030389,030461,060290,070320,091011,130219,170490,210220,210690,271019,271121,281810,283329,283429,283539,292249,292529,293299,293359,293399,293499,293626,293628,294110,294130,294190,300420,300490,320300,350300,350400,392020,392190,392310,392490,392630,392690,400932,401693,401699,420222,481029,481810,481910,481940,482110,551219,560392,560393,560811,611030,620333,620343,620433,620463,621133,630140,630710,630790,680229,710510,711311,730441,730690,730723,730791,730793,730799,730890,731021,731210,731815,732393,732399,732690,760421,760820,820559,830110,830210,830220,830241,830242,842123,842199,847290,847490,847989,848180,850434,850440,851220,853650,853669,853890,854370,854430,870829,870830,870840,870899,903289,910700,940169,940320,940360,940490,940599,950691,950790,960910,030617,130231,160529,283321,283524,292419,293629,310210,320412,392321,392610,420212,460219,481019,560790,621600,691110,700992,730721,731816,760429,761699,840991,841360,842139,842519,843149,848210,850490,853690,870810,870880,870893,900110,903180,903290,960820,210390,291631,292390,293329,294140,294150,330129,391690,391739,392410,420292,481820,580632,620453,630392,700910,701349,730439,730611,731582,731700,761510,830130,841391,848390,850433,853710,854449,030489,090412,110630,271012,283210,283531,293220,293369,400922,560729,560750,590310,600632,621143,650610,730729,731819,732620,841330,841370,848340,870894,871680,940592,960390,960810,030199,160419,190531,283650,291830,292429,293090,293319,293722,350691,391990,392062,392330,392390,620323,621710,810890,840999,841459,841480,842240,848360,870850,870870,870891,901720,030469,200989,293623,293625,293723,391740,392329,420239,481920,630231,630260,630539,650500,841221,843139,847780,848190,848410,853931,950300,960840,961000,282739,294120,391723,391731,392620,610990,630232,640299,701337,741220,841191,848310,848330,850710,851290,902620,940591,081090,220300,291529,320419,392010,392350,400911,482390,560391,610910,630720,731822,820551,830810,840734,842129,847410,848140,851140,940120,980400,220299,284920,291219,291711,293190,293339,293590,293621,340600,350699,390599,390761,391910,441990,610463,640419,691010,701090,721922,841350,842230,850152,853641,854411,854442,870322,870323,940350,030743,281119,283526,283630,293622,293627,293719,340212,382499,392113,442199,480300,481950,610433,700721,730539,730830,732490,830249,831130,841490,841869,848130,848250,850450,852721,853922,871160,871410,902610,940190,940390,940510,940540,293890,300320,392092,392099,420232,481013,590390,640399,680291,681099,700220,820600,821599,841590,841950,846729,848110,848420,850300,852990,853620,870892,210610,300390,310530,310540,340213,441919,482010,700711,721621,731824,830140,830710,830990,841430,842121,842131,842489,842890,847982,851762,940171,940330,940520,160432,200799,292250,340211,340290,392069,401519,540752,610333,660199,681389,721631,830120,830629,847790,847990,848350,854140,902519,902920,292800,293624,330499,390690,391390,391721,392640,570220,610462,620293,620342,701990,731029,732619,841451,848120,848490,851660,853720,854470,060314,230990,340220,590320,610342,610343,620462,621132,621142,680422,730419,731581,840890,841990,847160,848299,850131,851310,853110,854089,900490,961700,030359,300449,391810,400912,520839,560314,560819,610432,610520,620193,721720,731814,761090,841290,843143,851110,851679,930400,090411,200490,290613,300410,330410,350510,390769,392590,420221,560312,611020,701912,730661,761010,841381,841939,842833,842839,851230,851629,852859,854239,880320,940140,940161,980500,030343,200190,251690,270400,282619,291639,291814,292242,380210,382440,382600,390530,401120,442010,482369,540769,600192,700529,721921,846722,850212,853950,901580,901590,940180,940370,120770,120991,283660,284170,293139,330730,391000,391710,391729,392049,611595,620332,711319,711719,730630,730711,820110,848320,854460,870895,890399,901480,940389,940690,950659,950699,010611,051191,283421,291899,380892,381600,420231,611692,680610,720852,721012,721070,730719,820810,831110,841229,842531,847420,848790,850760,852580,853400,903033,090111,090220,283640,291736,310520,391890,400122,551299,600622,610332,621010,640391,680520,702000,720851,721420,721699,721934,730619,730640,730840,730900,731449,841989,842119,847330,847981,851190,851690,851830,853630,870380,901890,902710,940179,284990,291739,292241,390210,392119,400299,420291,620432,621210,630221,690220,690919,700719,700729,721049,721933,730210,731100,820750,820770,841510,843141,846719,850720,851821,853120,854390,901910,950490,961511,040721,170230,282300,284180,310221,320210,392043,401110,401140,420211,481190,560311,620322,680299,680510,720853,732510,760711,842951,842952,847439,854231,871200,902000,940429,282590,320611,401012,482370,610443,610510,620452,630622,640291,690721,720837,720890,721935,732119,732599,820719,841582,842511,843120,847130,847141,847170,848071,848291,850213,850421,850432,851712,851810,852190,852799,854330,870590,871499,910211,292121,310260,340130,391231,441090,441233,611011,620442,620443,621230,630533,670100,681140,690290,690390,721633,732611,760612,811299,820730,840290,840820,842720,842790,843041,850153,850811,850980,851829,852691,870193,940130,940430,950590,030792,290511,291539,321410,330790,350790,390720,390799,401180,570500,620192,620799,640340,680690,721410,721691,730531,732111,732410,847150,848060,850940,853321,853521,870410,871639,960329,110100,110900,151800,200979,270900,284390,290399,290619,310430,330491,340119,392112,441114,481830,610120,621040,640690,670210,720840,720854,730429,731441,810411,841440,841850,842611,842920,844130,846599,847149,851650,851770,860900,880240,901839,901920,950510,950629,960500,961900,030719,160521,251990,281511,284690,291712,390410,420100,441239,491199,590190,670490,691310,691410,701399,720838,720839,721041,721499,722090,732591,841940,841960,847480,851490,853590,870210,870423,870510,902820,190532,200600,220820,220830,252329,261400,280530,290544,293214,340239,340241,340242,340249,390469,390940,481099,550390,600539,610442,620140,640411,670419,681091,721061,721650,721710,722020,722830,731420,810399,821520,841620,841780,842132,843050,845530,851529,851713,851981,852589,853222,853224,854160,870121,870310,870822,902789,940199,940399,940511,940519,940542,940549,030749,080280,271111,271500,282630,283010,290391,293500,310510,380910,381800,382490,390760,441900,442190,470311,480269,500720,540784,620130,620230,640220,680293,690210,690722,720836,720916,720917,721230,721391,722540,730290,730449,730519,841112,841790,842930,842940,843031,843049,850422,851779,854143,854190,854320,854511,870360,870421,870911,880730,890190,902190,910221,940149,210120,220890,253010,290629,290930,320820,330300,620240,670290,720219,720918,730423,810419,811100,840219,843069,844180,845320,845490,845590,847950,851631,852862,852869,870290,870340,870540,880720,940440,030384,281820,320649,480593,540710,600410,600534,611420,640620,721912,721913,722599,730629,731431,840212,842911,850220,850423,852491,870194,870324,950450,970110,030349,080810,170290,200949,260111,282612,290960,381519,390740,440890,480261,480439,560130,570330,620212,681019,691200,720230,720810,730240,730590,740911,761490,844900,847529,848620,851632,854020,854142,890590,071333,090230,130232,170260,290129,290250,430310,441700,510820,620112,640510,680223,720449,721310,722012,722511,722550,731442,811239,846596,853949,890120,890392,940391,100610,110814,250830,251910,271320,280469,300220,382430,410320,481032,701959,710239,721990,722592,840410,843359,854519,860110,860699,870240,870460,880211,890322,890332,903020,910111,910121,030356,120600,230310,251749,270112,282570,290122,291221,520811,550410,611529,630630,630900,700510,730110,844140,852791,880230,890391,940141,970300,970600,030614,251710,281512,401011,720711,810430,811231,841181,847521,880212 |
| --- |
