# Supplementary material for: The impact of free trade port construction on regional import and export: Evidence from Hainan
Source: PLoS One. 2025 Aug 13;20(8):e0328875. doi: 10.1371/journal.pone.0328875 (PMC12349091; doi:10.1371/journal.pone.0328875)
Supplement: S1 File — S1 Table. HS codes of exported products. S2 Table. HS codes of imported products. S3 Table. RDD tests with polynomial fitting (bandwidth multiplier = 1.5). S4 Table. RDD tests with polynomial fitting (bandwidth multiplier = 2). S5 Table. Detailed parallel trend tests for LnExport. S6 Table. Detailed parallel trend tests for ExDensity. S7 Table. Detailed parallel trend tests for VarExport. S8 Table. Detailed parallel trend tests for LnImport. S9 Table. Detailed parallel trend tests for ImDensity. S10 Table. Detailed parallel trend tests for VarImport. (ZIP) [file pone.0328875.s001.zip › S2 Table. HS codes of imported products.docx]

**S2 Table. HS codes of imported products.**

Import

| 330499,260111,880240,270900,440122,290243,270112,271111,290511,271019,711319,290531,260300,910221,470321,253090,271012,470329,120510,270210,740311,080112,841112,271112,330300,270119,300490,420221,261510,330410,270730,382600,270111,420222,261000,851713,340130,910211,261400,120190,151190,910121,700220,100390,390740,240220,260112,640399,220820,271113,720712,890190,330491,720711,420291,740200,110814,260200,271320,210690,470500,121490,900410,381512,420292,880529,271311,880320,950890,151211,841191,290121,291739,300410,100790,270799,720918,851712,903289,610910,420330,270750,300420,252329,400280,720260,190110,390330,382319,903290,880330,640419,271500,390690,810520,080450,620140,901580,081060,870840,902190,260600,020230,350110,400110,420231,300432,841480,620193,040690,711719,720241,260800,847521,854370,290122,848180,910111,330420,890120,620240,420212,230120,890590,420211,261220,620293,720110,530500,220890,151530,170290,330590,151411,250300,611020,260400,841950,401693,520512,382370,030391,100630,230110,040510,711311,890392,270400,980500,230910,847720,610510,847982,220830,621210,621420,520511,730459,300449,130219,732690,910129,392690,030617,760110,550410,030636,870324,390319,330510,252310,620342,100119,420232,852990,390320,841360,901590,841221,611011,721391,620520,220421,040610,520100,711419,010611,847130,903180,300215,620292,250590,100199,851140,853890,841989,950300,901420,250100,010229,722490,040210,640359,030390,847989,020442,330210,510111,910229,970600,640299,854430,620211,842230,620192,880212,040221,902750,761610,382499,180690,250510,880730,890332,400122,300450,842199,293499,340239,750210,761699,830210,591132,620442,390190,261690,853690,902620,841181,081090,720310,470311,382490,852610,640391,731829,620220,120740,890322,902221,900150,890690,250700,854142,293190,440719,390390,847780,620230,851762,842123,293890,760612,090111,842139,190190,071310,392051,230240,852910,420219,620130,020220,851110,620462,870323,842890,841459,390210,851010,290124,381511,702000,902610,853650,690740,901120,120400,890391,620343,284690,611030,290220,902519,730441,854442,020714,940190,731815,848210,902000,847990,330749,847149,071410,902720,841199,100590,620291,270820,390769,294190,850980,650500,853710,902780,151329,010221,850440,880720,381590,630790,620630,350400,100640,390610,810890,640319,030627,080261,902781,392590,720720,271114,710239,870829,620120,040410,731822,841350,850730,902590,180632,080390,390140,390130,382100,611420,390110,820730,842489,850490,960329,830249,290129,151620,390230,420310,847790,710692,843360,390290,848340,902730,620444,854190,591131,392620,902139,731816,842119,880211,621410,841869,340600,080111,220299,750890,170490,610990,841590,440349,621010,200989,848130,281119,960830,852859,392010,848140,620191,293399,848190,830160,140490,400121,850760,854239,210111,151800,722790,820559,200899,620449,340211,690390,030616,230990,901890,843991,620443,853720,961900,847330,520514,290532,520524,853921,848120,284520,740400,820890,080929,251320,851150,890710,030389,401699,902214,281820,150810,610822,950450,041000,730890,292610,030313,620441,440712,230230,847160,842220,220600,390120,830140,611012,890399,640420,852691,560750,080299,240210,400129,842542,902710,720230,903149,853641,010612,440321,848790,851770,440290,851180,730729,252922,050400,940540,230320,847141,731290,853110,400270,842240,611120,290110,290545,620341,440711,790111,330730,620463,850140,121190,841370,902920,180631,261390,840219,410150,392310,848330,392062,340111,902789,190230,300439,853180,121221,620452,293359,848410,281210,841290,710310,851671,731824,940110,690220,252620,901839,901910,841490,401695,850131,848310,480525,731512,391732,848390,621111,854140,880220,711620,842410,620590,030214,870830,210112,902680,843999,740100,620469,020329,740319,848060,190531,852349,940592,900490,851660,230630,440710,901210,847590,481420,390730,843880,842839,400231,620459,841229,850151,940599,848110,330290,841990,620311,290941,620451,901850,903190,121120,842121,851190,701890,851631,760120,230641,903210,620610,391731,030743,841391,281000,901110,110630,620461,842129,610210,890331,293090,290321,843139,630720,621710,841620,081340,852589,731100,440714,841939,853224,760900,901812,851680,270300,620453,901490,190590,620113,621132,620213,850450,961519,410320,620111,480261,460219,041090,440391,620331,380210,620349,844332,081190,842490,640411,701939,200799,901849,820590,901820,961511,940199,903220,290399,852351,400220,910212,040590,390469,020430,960810,851989,391740,220410,340220,293349,845961,842820,847170,901480,901060,847730,820600,020441,843359,843910,293722,903281,290719,620299,640351,851521,700711,220429,251749,621440,481019,902300,761300,620332,846023,320420,610712,842290,611490,040229,853669,846024,220300,851629,620530,620432,440399,842860,732020,681599,290723,711790,851830,281219,722540,843680,291439,848360,961620,611710,380992,841381,480524,170199,380894,840290,902790,261610,400219,854390,851130,841231,722511,701990,850300,611130,340399,260900,902212,330790,610590,620333,700721,120242,871680,900211,281122,520523,591000,392390,121229,854449,621142,850431,902219,080280,030619,870340,610342,903300,611019,391400,381519,481940,294150,730449,842511,848420,853190,853221,252100,040900,732490,391810,610711,481022,520522,848620,350190,841410,842790,330610,843290,611090,830710,320414,491110,190532,090411,020649,610610,230310,611430,841451,902580,392190,730840,841932,260700,291619,391990,854233,851610,842099,440323,620640,843351,890790,481190,854160,080290,940549,020629,292429,200600,621133,040150,610821,846490,842191,051191,620290,340213,841920,330720,060220,290949,730900,846596,710510,440729,293369,120600,481920,160420,294000,480255,470319,903110,820900,610520,520513,843810,841320,420500,761090,210390,200931,842010,970110,902229,846620,620212,850410,392220,842420,440220,961590,610442,321490,480519,850162,280461,650699,844190,940421,150290,620439,290230,400239,848299,730640,847150,848079,854143,940490,843780,847180,442199,847920,851529,730661,831000,570500,030462,401610,902990,854129,620433,854890,691110,220870,902690,854231,853340,940360,940161,030520,321290,901510,970300,846592,843340,900130,400911,400211,911390,850152,851822,151790,381600,842430,040120,300670,080430,850940,230660,950629,730722,721119,620431,251010,940591,850590,480411,711711,090510,621490,382000,903089,200819,440713,950691,841330,853540,382200,080540,382219,640220,910219,320890,481960,590700,621510,621143,853649,961210,901813,261100,482340,841981,580410,900190,740911,710391,252910,840490,350220,610230,350510,440799,903084,848490,300440,391739,520532,400941,846593,843143,290722,844319,030621,851769,900110,842611,870422,441990,150420,900319,252210,842833,210500,392063,901380,080131,610462,853939,852290,850811,293629,401691,401519,853661,620339,391239,820412,961700,391910,851810,130190,846299,890319,731823,843110,030392,081040,851679,852491,340250,610120,680291,903010,621139,080262,940350,470100,910400,294200,510121,391722,852580,950699,853321,830810,901180,710691,030633,903120,290919,520612,391890,220860,320990,710399,051000 |
| --- |
