# Supplementary material for: The impact of free trade port construction on regional import and export: Evidence from Hainan
Source: PLoS One. 2025 Aug 13;20(8):e0328875. doi: 10.1371/journal.pone.0328875 (PMC12349091; doi:10.1371/journal.pone.0328875)
Supplement: S1 File — S1 Table. HS codes of exported products. S2 Table. HS codes of imported products. S3 Table. RDD tests with polynomial fitting (bandwidth multiplier = 1.5). S4 Table. RDD tests with polynomial fitting (bandwidth multiplier = 2). S5 Table. Detailed parallel trend tests for LnExport. S6 Table. Detailed parallel trend tests for ExDensity. S7 Table. Detailed parallel trend tests for VarExport. S8 Table. Detailed parallel trend tests for LnImport. S9 Table. Detailed parallel trend tests for ImDensity. S10 Table. Detailed parallel trend tests for VarImport. (ZIP) [file pone.0328875.s001.zip › S7 Table. Detailed parallel trend tests for VarExport.docx]

**S7 Table. Detailed parallel trend tests for VarExport.**

| Parallel trend test of VarExport | | | | | | |
| --- | --- | --- | --- | --- | --- | --- |
| VarExport. | $Coef.$ | Robust.Std.Err. | $t$ | $P>\vert t\vert$ | [95% Conf. Interval] | |
| pre_14 | $0.750447$ | $0.603027$ | $1.24$ | $0.214$ | $-0.43424$ | $1.935132$ |
| pre_13 | $-0.04759$ | $0.585731$ | $-0.08$ | $0.935$ | $-1.1983$ | $1.103113$ |
| pre_12 | $-0.70251$ | $0.585497$ | $-1.2$ | $0.231$ | $-1.85276$ | $0.447732$ |
| pre_11 | $-0.47642$ | $0.609414$ | $-0.78$ | $0.435$ | $-1.67365$ | $0.720814$ |
| pre_10 | $-0.03155$ | $0.616014$ | $-0.05$ | $0.959$ | $-1.24175$ | $1.178645$ |
| pre_9 | $0.125524$ | $0.601458$ | $0.21$ | $0.835$ | $-1.05608$ | $1.307127$ |
| pre_8 | $0.267694$ | $0.599489$ | $0.45$ | $0.655$ | $-0.91004$ | $1.445427$ |
| pre_7 | $0.167582$ | $0.560891$ | $0.3$ | $0.765$ | $-0.93432$ | $1.269488$ |
| pre_6 | $-0.36699$ | $0.567655$ | $-0.65$ | $0.518$ | $-1.48219$ | $0.748204$ |
| pre_5 | $-0.71354$ | $0.548985$ | $-1.3$ | $0.194$ | $-1.79206$ | $0.364975$ |
| pre_4 | $-0.43043$ | $0.543229$ | $-0.79$ | $0.429$ | $-1.49763$ | $0.636781$ |
| pre_3 | $-0.86154$ | $0.448517$ | $-1.92$ | $0.055$ | $-1.74268$ | $0.019599$ |
| pre_2 | $-0.32473$ | $0.341865$ | $-0.95$ | $0.343$ | $-0.99643$ | $0.346887$ |
| current | $1.895962$ | $0.453983$ | $4.18$ | $0$ | $1.004083$ | $2.78784$ |
| post_1 | $1.475388$ | $0.595098$ | $2.48$ | $0.013$ | $0.30628$ | $2.644496$ |
| post_2 | $0.676704$ | $0.627699$ | $1.08$ | $0.282$ | $-0.55645$ | $1.909859$ |
| post_3 | $-1.75901$ | $0.568987$ | $-3.09$ | $0.002$ | $-2.87682$ | $-0.6412$ |
| post_4 | $-2.50344$ | $0.572766$ | $-4.37$ | $0$ | $-3.62868$ | $-1.37821$ |
| post_5 | $-2.472$ | $0.554485$ | $-4.46$ | $0$ | $-3.56132$ | $-1.38267$ |
| post_6 | $-2.74854$ | $0.554511$ | $-4.96$ | $0$ | $-3.83791$ | $-1.65917$ |
| post_7 | $-2.80457$ | $0.562531$ | $-4.99$ | $0$ | $-3.9097$ | $-1.69944$ |
| post_8 | $-3.50184$ | $0.578768$ | $-6.05$ | $0$ | $-4.63887$ | $-2.36481$ |
| post_9 | $-4.47759$ | $0.53722$ | $-8.33$ | $0$ | $-5.53299$ | $-3.42219$ |
| post_10 | $-4.78894$ | $0.549661$ | $-8.71$ | $0$ | $-5.86879$ | $-3.7091$ |
| post_11 | $-4.63609$ | $0.530878$ | $-8.73$ | $0$ | $-5.67903$ | $-3.59314$ |
| post_12 | $-4.34483$ | $0.55425$ | $-7.84$ | $0$ | $-5.43369$ | $-3.25598$ |
| post_13 | $-4.85287$ | $0.524368$ | $-9.25$ | $0$ | $-5.88302$ | $-3.82271$ |
