# Supplementary material for: The impact of free trade port construction on regional import and export: Evidence from Hainan
Source: PLoS One. 2025 Aug 13;20(8):e0328875. doi: 10.1371/journal.pone.0328875 (PMC12349091; doi:10.1371/journal.pone.0328875)
Supplement: S1 Appendix — (DOCX) [file pone.0328875.s002.docx]

Appendix Table 1. Major Trading Partners and Total Trade Volume (USD)

| **Partner Code** | **Partner Name** | **Total Trade Volume (USD)** |
| --- | --- | --- |
| 502 | United States | 586,979,636,456 |
| 116 | Japan | 317,282,958,519 |
| 133 | South Korea | 285,580,741,898 |
| 110 | Hong Kong, China | 279,558,186,869 |
| 143 | Taiwan, China | 260,615,359,263 |
| 141 | Vietnam | 192,290,082,708 |
| 304 | Germany | 191,918,837,811 |
| 601 | Australia | 171,162,255,057 |
| 122 | Malaysia | 131,475,736,085 |
| 410 | Brazil | 120,470,998,684 |
| 344 | Russia | 108,189,145,471 |
| 136 | Thailand | 98,653,976,767 |
| 303 | United Kingdom | 92,433,909,108 |
| 309 | Netherlands | 91,795,649,022 |
| 132 | Singapore | 89,244,193,188 |
| 111 | India | 87,697,016,888 |
| 112 | Indonesia | 78,463,077,472 |
| 131 | Saudi Arabia | 67,165,151,536 |
| 305 | France | 66,651,212,349 |
| 501 | Canada | 64,157,931,135 |
| 129 | Philippines | 61,217,180,198 |
| 429 | Mexico | 61,045,482,560 |
| 307 | Italy | 55,164,260,879 |
| 138 | United Arab Emirates | 49,365,167,079 |
| 412 | Chile | 45,267,549,016 |
| 312 | Spain | 37,904,449,314 |
| 244 | South Africa | 36,063,670,276 |
| 327 | Poland | 31,051,179,225 |
| 114 | Iraq | 30,226,745,930 |
| 301 | Belgium | 28,580,195,201 |
| 137 | Turkey | 24,077,893,084 |
| 434 | Peru | 23,601,217,044 |
| 331 | Switzerland | 22,422,698,455 |
| 145 | Kazakhstan | 21,508,234,780 |
| 236 | Nigeria | 19,272,872,688 |
| 106 | Myanmar | 18,894,320,252 |
| 352 | Czech Republic | 18,871,225,677 |
| 126 | Oman | 18,735,931,998 |
| 609 | New Zealand | 18,129,149,863 |
| 306 | Ireland | 18,041,113,159 |
| 330 | Sweden | 17,886,438,720 |
| 115 | Israel | 17,539,208,417 |
| 127 | Pakistan | 17,482,544,587 |
| 202 | Angola | 16,505,884,799 |
| 103 | Bangladesh | 15,875,451,539 |
| 113 | Iran | 14,933,394,317 |
| 347 | Ukraine | 14,879,762,749 |
| 215 | Egypt | 14,551,325,502 |
| 118 | Kuwait | 14,282,932,583 |
| 402 | Argentina | 13,898,270,619 |
